# Genome Sequence of the Historical Clinical Isolate *Burkholderia pseudomallei* PHLS 6

**DOI:** 10.1128/genomeA.00649-16

**Published:** 2016-06-30

**Authors:** Patrik D’haeseleer, Shannon L. Johnson, Karen W. Davenport, Patrick S. Chain, Joe Schoeniger, Debjit Ray, Anupama Sinha, Kelly P. Williams, José Peña, Steven S. Branda, Sahar El-Etr

**Affiliations:** a Biosciences and Biotechnology Division, Lawrence Livermore National Laboratory (LLNL), Livermore, California, USA; b Los Alamos National Laboratory (LANL), Los Alamos, New Mexico, USA; c Sandia National Laboratories (SNL), Livermore, California, USA

## Abstract

Here, we present the draft genome sequence of *Burkholderia pseudomallei* PHLS 6, a virulent clinical strain isolated from a melioidosis patient in Bangladesh in 1960. The draft genome consists of 39 contigs and is 7,322,181 bp long.

## GENOME ANNOUNCEMENT

*Burkholderia pseudomallei* is a Gram-negative pathogenic saprophyte endemic to Southeast Asia, India, and northern Australia (1, 2). It is the causative agent of melioidosis, a potentially fatal infectious disease with a wide spectrum of symptoms (3). Melioidosis mortality rates range from 14% to 40% (4, 5), exceeding 70% without treatment with effective antimicrobials (6, 7). The global number of melioidosis cases per year has been estimated to be around 165,000, including 89,000 deaths (2). *B. pseudomallei* is on the CDC category B list (8, 9) and is considered to be a potential bioweapon (10) due to its prevalence in soil, multiple routes of infection, low infectious dose, high mortality rates, native resistance to a wide range of antibiotics and disinfectants, and lack of an effective vaccine.

Like other *Burkholderia* species, *B. pseudomallei* strains exhibit a remarkable level of genomic plasticity (11–16). The high abundance of simple sequence repeats (SSRs) in *Burkholderia mallei* and *B. pseudomallei* strains drives rapid genomic adaptation (17), even during the course of acute infection in a single patient (18–20) or short-term passaging *in vitro* (20, 21). *B. pseudomallei* PHLS 6 was chosen for sequencing because it is a virulent clinical strain that has undergone minimal lab manipulation. It was originally isolated from a melioidosis patient in Bangladesh in 1960. The PHLS 6 strain was also found to be highly efficient at entering and surviving inside amoebae, a mechanism thought to play a role in the prevalence of *B. pseudomallei* in soils (22).

Genomic DNA was isolated using the Norgen bacterial genomic DNA isolation kit and initially sequenced independently using Illumina technology at Sandia National Laboratories (SNL) and Los Alamos National Laboratory (LANL). SNL sequence data consisted of 10,692,593 NextSeq 150-bp paired-end (PE) reads of short inserts (Nextera DNA library prep kit) and 3,500,043 reads of long inserts (Nextera mate-pair prep kit) trimmed with NxTrim (23). Assembly with SPAdes, Bridger, and Gap Filler (24–26) yielded 40 scaffolds with 220 contigs. LANL sequence data consisted of 15,757,418 MiSeq 250-bp PE reads of short inserts (300 ± 70 bp) (NEBNext Ultra DNA library prep), which were trimmed and filtered for quality and reduced to a total of 300× genome coverage for use in the assemblies. Assemblies from IDBA and Velvet were merged with parallel Phrap (27–30), producing 70 contigs. Because of the high number of repeats in the genome, it was decided to combine the SNL long-insert data and LANL short-insert data using ALLPaths (31) and merge the resulting assembly with the prior SNL and LANL assemblies in parallel Phrap to generate the final improved draft assembly, with 39 contigs totaling 7,322,181 bp (G+C content, 68.1%). Annotation utilized an LANL in-house Ergatis workflow with minor manual curation (32) and found 6,156 coding regions, 11 rRNA sequences, and 59 tRNA sequences. The genome contains 118 genes associated with antibiotic and toxic compound resistance, as well as 16 genes associated with cellular invasion and intracellular resistance (33).

### Nucleotide sequence accession numbers.

This whole-genome shotgun project has been deposited at DDBJ/ENA/GenBank under the accession no. LWRR00000000. The version described in this paper is version LWRR01000000.
